# Thienylene combined with pyridylene through planar triazine networks for applications as organic oxygen reduction reaction electrocatalysts†

**DOI:** 10.1039/d3ra01431b

**Published:** 2023-04-17

**Authors:** Kosuke Sato, Nodoka Osada, Hidenori Aihara

**Affiliations:** a Organic Materials Chemistry Group, Sagami Chemical Research Institute 2743-1 Hayakawa Ayase Kanagawa 252-1193 Japan kosuke-sato@sagami.or.jp; b Course of Applied Science, Graduate School of Engineering, Tokai University 4-1-1 Kitakaname Hiratsuka Kanagawa 259-1292 Japan

## Abstract

Covalent triazine networks are interesting candidates for organic electrocatalytic materials due to their tunable, durable and sustainable nature. However, the limited availability of molecular designs that ensure both two-dimensionality and functional groups in the π-conjugated plane has hindered their development. In this work, a layered triazine network composed of thiophene and pyridine ring was synthesized by the novel mild liquid phase condition. The resulting network showed layered nature since its intramolecular interaction stabilized its planar conformation. The connection on the 2-position of the heteroaromatic ring prevents steric hindrance. The simple acid treatment method could be used to exfoliate the networks, resulting in high yields of nanosheets. The planar triazine network showed superior electrocatalytic properties for the oxygen reduction reaction in the structure-defined covalent organic networks.

## Abstract 2

Asymmetric, low-crystalline, D–π–A type layered triazine networks was exfoliated to nanosheets and used as metal-free electrochemical ORR catalyst.

## Graphical abstract

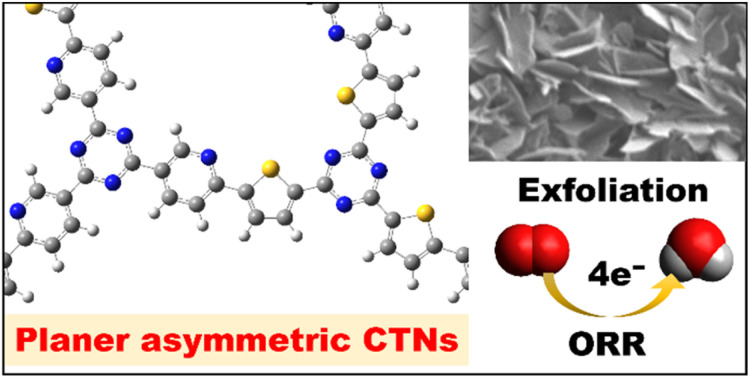

## 1. Introduction

**Fig. 1:**
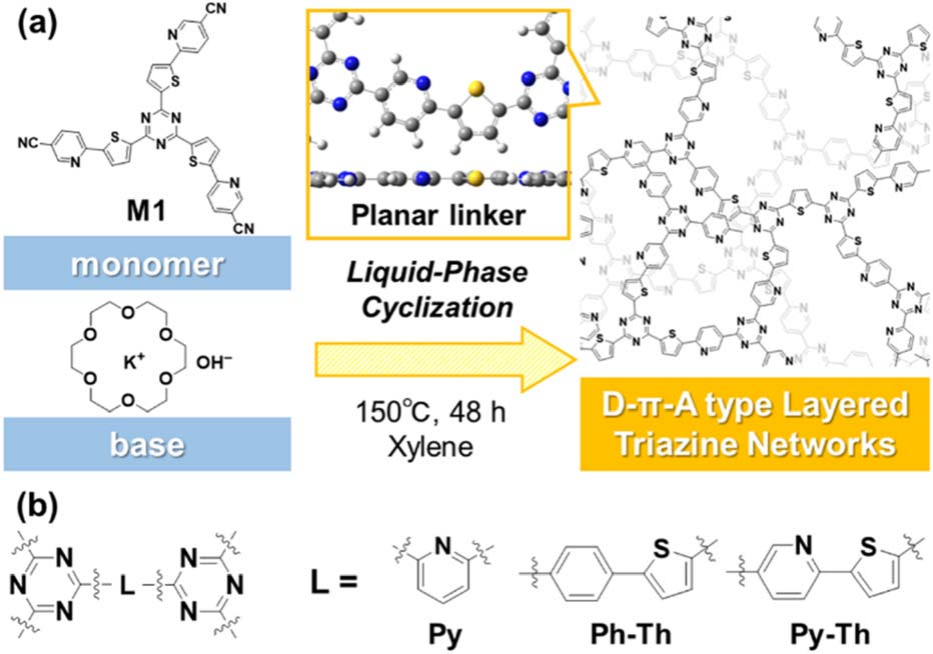
(a) Schematic representation of the liquid-phase cyclization for the synthesis of amorphous layered triazine networks. (b) Molecular structures of the triazine networks synthesized in the present study.

Covalent triazine frameworks have potential because of their properties and the emergence of new functions originating from their extended π-conjugated structures.^1–5^ The two-dimensional (2D) nature enhances their properties, in contrast with the amorphous covalent triazine networks (CTN) with a shorter π-conjugation length.^6–8^ However, the characteristic 2D structure of the frameworks is ensured by the limited small linker moieties between the triazine rings. If a versatile strategy to design 2D structured CTN is revealed, a variety of functional units can be embedded in the long-range π-conjugated plane and can contribute to the production of fascinating applications. Herein, we report the design and synthesis of layered CTN and their exfoliation into nanosheets under mild conditions (Fig. 1). The layered CTN bearing pyridylene-thienylene linkers (Py–Th) exhibited enhanced electrocatalytic activity for the oxygen reduction reaction (ORR) in the structure-defined covalent organic networks.

Recently, various electrochemical ORR catalysts have been studied as alternative noble metal-based electrocatalysts. Covalent organic network polymers have attracted attention as interesting electrocatalysts, owing to their sustainability, low solubility to solution, and structural tunability.^9–13^ In particular, π-conjugated network polymers comprising heteroaromatic rings present great potential for organic ORR electrocatalysts.^14–23^ The electronegative nitrogen and electropositive carbon atom at appropriate distances contribute to the multi-point interactions with oxygen.^21,22^ Considering that planarity is a key factor that ensures smooth charge transfer and high interface area, 2D covalent organic frameworks are potential candidates as superior organic electrocatalysts.^19–23^ However, only a limited number of highly crystalline CTN have been reported because symmetry and flatness are required for the linker moiety between triazine rings.^6–8,24,25^ The development of a strategy to obtain layered CTN can facilitate the unprecedented combination of the functional groups as novel 2D triazine networks. Here, we synthesized layered CTN containing both electron donor units and catalytic active sites to enhance the electrocatalytic properties.

The exfoliation of layered materials is an effective method for obtaining 2D materials.^26–28^ Organic layered compounds comprising layers stacked *via* van der Waals interaction such as carbon-related materials, covalent organic frameworks, and layered network polymers are exfoliated into nanosheets in the liquid phase with the application of certain stimuli.^29–37^ In previous studies, several methods such as liquid-phase, mechanical, and charged exfoliation have been applied to the triazine frameworks.^31–36^ The periodic highly crystalline structure in the lateral direction reinforces vertical interlayer interaction; therefore, the exfoliation of the triazine frameworks requires harsh conditions. The present study prepared low-crystalline 2D structured CTN to facilitate exfoliation. In our previous study, amorphous layered conjugated-polymer networks were reported as effective organic electrocatalysts for water splitting.^37^ The amorphous layered structure increased the interface area and facilitated charge transportation. Herein, we report the synthesis of amorphous layered CTN for ORR electrocatalysts. The planar conformation of the heteroaromatic linker is a key factor for the 2D nature. The resultant low-crystalline CTN were efficiently exfoliated into nanosheets in mild liquid phase. Moreover, the resultant layered Py–Th was applied to a metal-free electrocatalyst for ORR.

## 2. Materials and methods

### 2.1 Synthesis of Py–Th

Under air, potassium hydroxide (1.9 mg, 0.033 mmol) and 18-crown-6 ether (8.5 mg, 0.033 mmol) was dissolved in ethanol (1 mL) and stirred for 10 min. The solution was concentrated to remove solvent under reduced pressure to give an oil product. To this oil, M1 (500 mg, 0.80 mmol) and xylene (0.8 mL) were added. The mixture was refluxed at 150 °C for 48 h. After cooling to the room temperature, the crude product was corrected by filtration. The crude product was purified by washing with xylene, CHCl_3_ and water to give Py–Th as yellow solid (405 mg, 81%).

### 2.2 Characterization

The molecular structure was analyzed by Fourier transform infrared (FT-IR) spectroscopy using attenuated total reflection method (Jasco, FT-IR 4200). The light absorption properties were measured by ultraviolet-visible (UV-vis) spectrometer (Jasco, UV-vis 770) with diffuse reflection method. The bonding states were estimated by X-ray photoelectron spectroscopy (XPS, outsourced analysis). The pelletized CTN samples were measured. The ^1^H-NMR chart in CDCl_3_ solution is obtained by nuclear magnetic resonance (NMR, Bruker, Ascend 400). The ^13^C-NMR charts in solid states were obtained by cross polarization/magic angle spinning method (outsourced analysis). Assemble structure was analyzed by X-ray diffraction (XRD) with Cu-Kα radiation (Rigaku, Smart Lab). The powdered sample was set on a silicon sample holder without diffraction peaks in the measured range. The morphologies of the CTN samples were observed using a field-emission type scanning electron microscopy (SEM, JEOL, JSM 7100 F). The dispersion liquid containing the exfoliated CTN was dropped on a heated copper foil for SEM observation.

### 2.3 Calculation and simulation

DFT calculations to study the stable conformation of the CTN performed based on B3LYP-D3BJ/6-31G++(d,p) level theory by using Gaussian 16. Model compounds containing around 70 atoms were optimized on that condition for a simplicity. In XRD chart simulation, periodic 2D cartesian of Py–Th was optimized on B3LYP/6-31G(d) level theory. The 3D coordinate was constructed by laminating the 2D cartesian with interlayer distance according to the literature.^1,6^

### 2.4 Electrochemical measurements

A three-electrode setup in a beaker cell was used for electrochemical measurements. A grassy carbon rod (3 mm of diameter) and Ag/AgCl electrode with saturated KCl solution were used as a counter electrode and reference electrode. The catalyst film coated ring-disk electrode (RDE, grassy carbon, 5 mm in diameter) was used as the working electrode. The catalyst film was consisting of the CTN, conductive carbon (Vulcan XC-72R, Cabot) and Nafion (purchased from Aldrich). Typically, the mixture of Py–Th/carbon was prepared *via* polymerization of M1 in a solution containing 9 times the weight of the conductive carbon. To obtain the catalyst ink, the Py–Th/carbon mixture (5 mg) and Nafion solution (0.05 mL) were dispersed in ethanol (0.95 mL) with sonication. The catalyst ink (35 μL) was casted to the RDE and dried at 60 °C to fabricate the catalyst film. Conductive carbon without the CTN and Pt/C (10 wt%, Aldrich) were used as reference. Detailed setups were described in the ESI.†

## 3. Result and discussion

### 3.1 Material synthesis and characterization

The triazine networks, Py–Th, were synthesized by a base-catalyzed cyclization of the trinitrile monomer (M1). M1 was synthesized from commercially available reagents *via* three steps (Scheme S1 in the ESI†). The liquid-phase polymerization of M1 was performed in xylene using KOH/18-crown-6 ether to activate the nitrile group. Py–Th was obtained in 81% yield. To the best of our knowledge, this is the first report on the base-catalyzed liquid-phase polymerization of nitrile groups, in contrast to reported acid-catalyzed cyclization to construct triazine networks.^2–5,38,39^ When the nitrile monomer contains basic groups such as pyridines and amines, the base-catalyzed approach, under mild liquid-phase conditions, is more effective than the acid-catalyzed trimerization method.

**Fig. 2:**
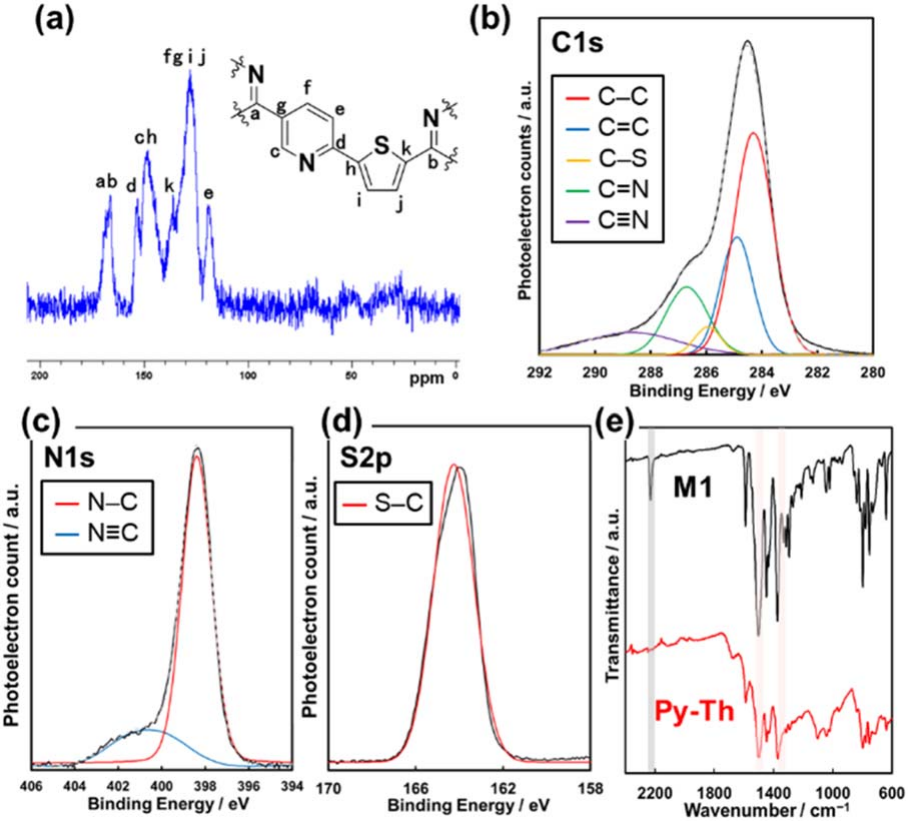
(a) Solid-state nuclear magnetic resonance (NMR) chart for Py–Th. (b–d) X-ray photoelectron spectroscopy (XPS) spectra of Py–Th focusing on C 1s, N 1s, and S 2p. (e) Fourier transform infrared (FT-IR) spectra of M1 and Py–Th.

The resultant product was identified to be Py–Th by solid-state NMR, XPS, and FT-IR absorption spectroscopy (Fig. 2a–e). The attribution of NMR signals was performed according to the comparison of the experimental and simulated charts (Fig. 2a and S1†). The experimental and simulated charts were similar; therefore, the NMR chart shows the generation of Py–Th. The XPS spectra show the bonding states of the carbon, nitrogen, and sulfur atoms. The C 1s XPS spectrum was fitted into five-carbon species at the binding energies of 284.1, 284.9, 285.8, 286.5, and 288.4 eV, corresponding to the C–C, C=C, S–C, N=C–N, and C≡N groups, respectively (Fig. 2b). The C≡N group observed on the Py–Th sample originated from terminal groups. The photoelectron signals attributed to N 1s and S 2p were detected from the resultant sample (Fig. 2c and d). The N 1s spectrum was fitted into two nitrogen species at the binding energies of 398.2 and 400.7 eV attributed to the C=N–C and C≡N groups, respectively. The FT-IR spectra revealed the formation of Py–Th (Fig. 2e and S1†). The resultant polymer sample exhibited the absorption bands of the triazine-ring stretching vibration at 1355 and 1503 cm^−1^ and aromatic C=C stretching vibration at 1404 and 1445 cm^−1^. The triazine derived absorption peaks were slightly shifted from 1347 and 1496 cm^−1^ of M1 due to appearance of tri-thienyl triazine moiety. In addition, the absorption bands were broader than those of the monomer during the polymerization. The absorption at 2210 cm^−1^ derived from the stretching vibration of C≡N groups reduced after the polymerization. These spectroscopies indicated the formation of Py–Th by liquid-phase polymerization. Py and Ph–Th were synthesized and characterized as reference samples (Schemes S2, S3 and Fig. S2†).

### 3.2 Assembled structures

**Fig. 3:**
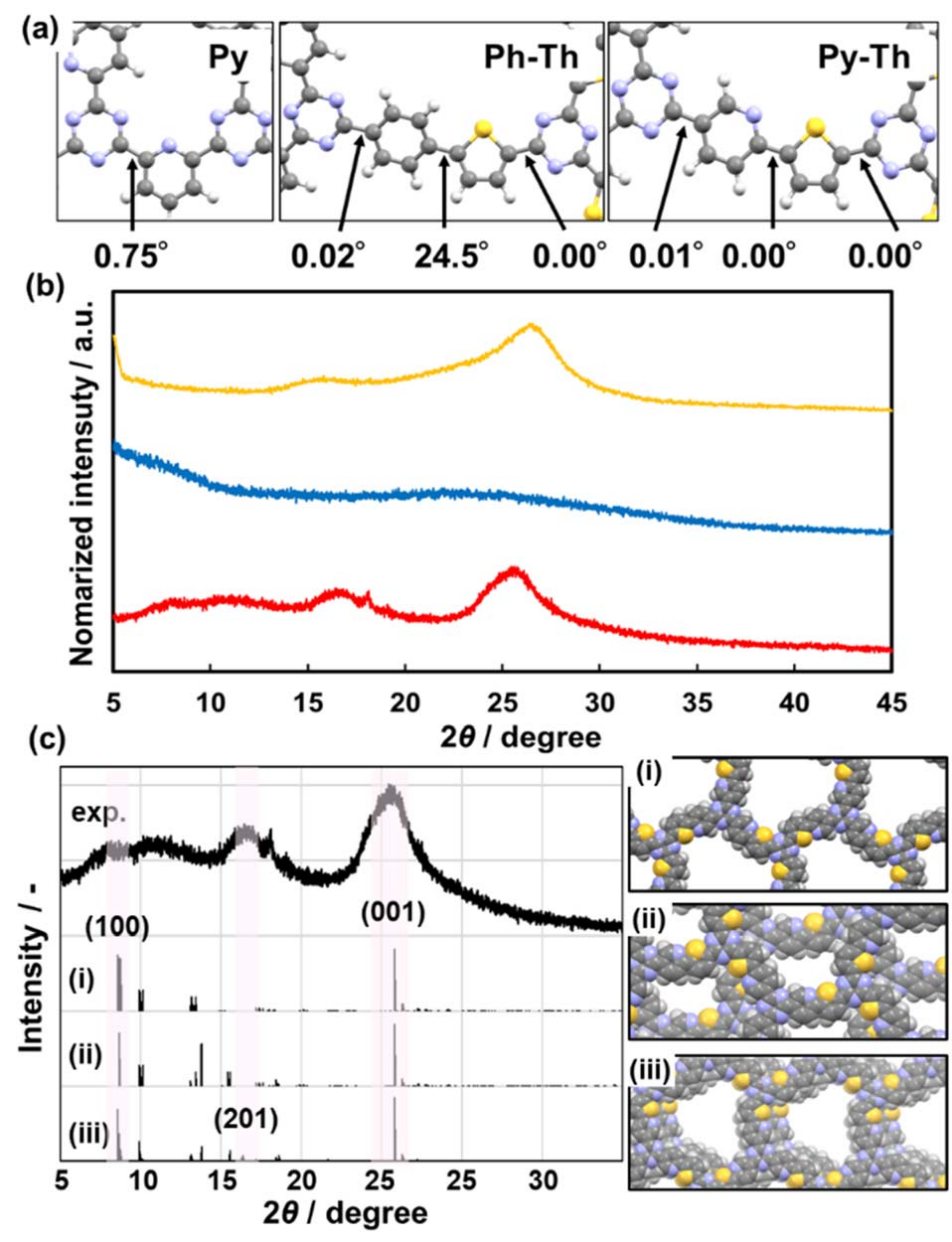
(a) Dihedral angles of the optimized structures calculated by using the DFT method. (b) XRD profiles of Py, Ph–Th, and Py–Th (yellow, blue, and red lines, respectively). (c) Comparison of experimental and simulated XRD charts of Py–Th. The stacking states corresponding to eclipsed (i), staggered (ii) and partially slipped (iii) displacement with (0, 0), (0.5, 0.5) and (0.2,0.2) of offset, respectively.

The stable conformation of Py–Th was calculated using the density functional theory (DFT) method based on the B3LYP+D3BJ/6-31+G(d,p) level theory (Fig. 3a). The model compound corresponding to Py–Th was optimized to a completely flat nature with practically 0° of dihedral angles. The angle scan profile of the dihedral angle between the pyridine and thiophene rings shows that Py–Th exhibited two different stable planar conformations (Fig. S3†). The stabler conformation appeared to be stabilized by the strong intramolecular interaction between the nitrogen and sulphur atoms.^40^ The molecular design of Py–Th can realize a planar primal layer comprising multiple rings. The connection on the 2-position of the heteroaromatic ring prevents steric hindrance between the C–H bonds of adjacent linkers. The strategy was effective in the design of 2D organic networks.

The X-ray diffraction (XRD) results indicated that Py–Th formed a roughly stacked layered structure (red line in Fig. 3b). Py–Th exhibited broadened weak peaks around 2*θ* = 16.8° and 25.9°, corresponding to the lattice spacings of 5.3 and 3.4 Å, respectively. The periodic space around 3.4 Å corresponded to the interlayer distance of π–π stacking.^1,39^ The broadened diffraction peak indicated that the interlayer distance of Py–Th had a broad distribution centered at 3.4 Å. The periodic space around 5.3 Å was ascribed to the weak periodicity in the planar network comprising heteroaromatic rings. According to the comparison of the experimental and simulated charts, Py–Th containing the partially slipped stacking (Fig. 3c). Three types of A-B stacking state displaced from next layer were considered. The partially slipped stacking displaced by (0.2, 0.2) of offset explain the periodic space around 5.3 Å. Furthermore, the broaden diffraction in range of 2*θ* = 5–20° should be derived from various slipped stacking states. Therefore, Py–Th have periodicity in laminated direction but low periodicity in lateral direction.

Py, classified as a typical 2D triazine frameworks, exhibited diffraction peaks around 2*θ* = 16.6° and 26.5° (yellow line, Fig. 3b). Py and Py–Th exhibited similar low-periodic layered structures. Contrarily, Ph–Th did not exhibit diffraction peaks in the XRD charts (blue line, Fig. 3b). The Ph–Th polymer was in an isotropic amorphous state because of the twisted nature derived from the distortion between the benzene and thiophene rings. The simulated dihedral angle of Ph–Th was 24.5° for the benzene–thiophene bond (Fig. 3a). The lack of flatness resulted in the amorphous nature of Ph–Th. We expect that the proper molecular design toward a planar conformation will enable triazine networks to assemble a layered structure although the linkers comprise multiple functional aromatic rings.

### 3.3 Macroscopic morphology and exfoliation

**Fig. 4:**
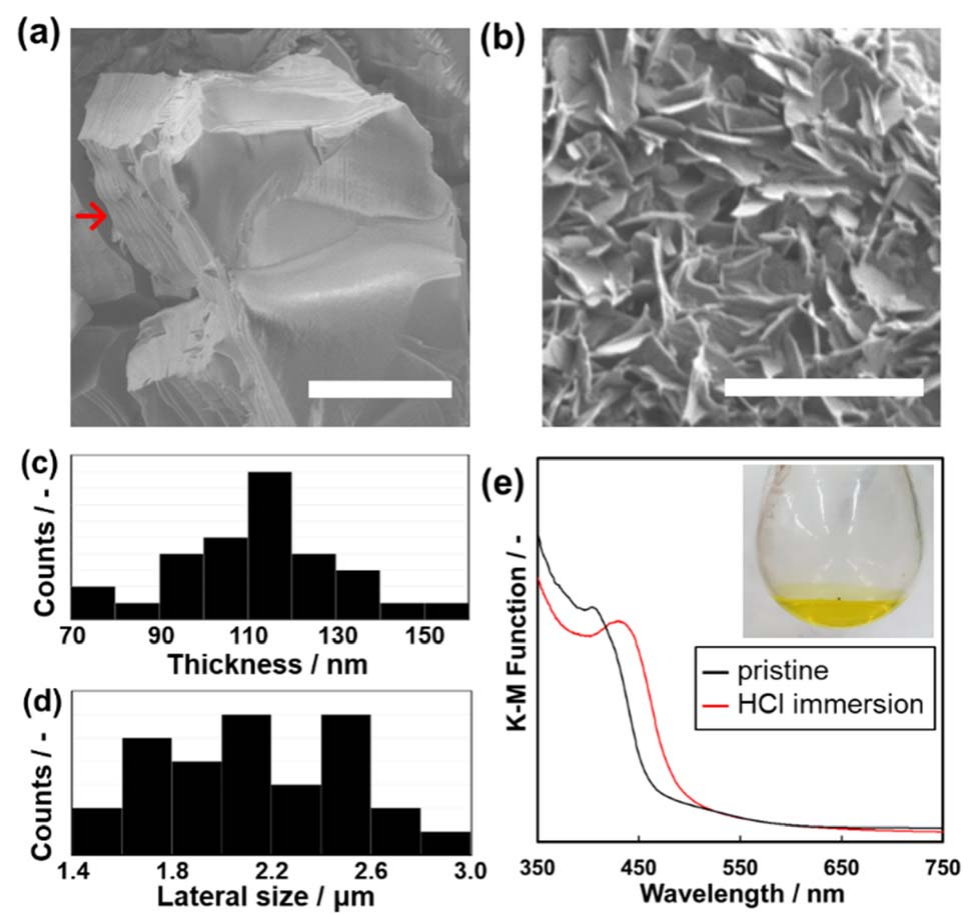
(a) Scanning electron microscopy (SEM) image of the pristine Py–Th particle (scale bar, 5 μm). (b) SEM image of the exfoliated Py–Th nanosheets (scale bar, 5 μm). (c and d) Thickness (c) and lateral (d) size histograms of the nanosheets. (e) Ultraviolet-visible (UV-Vis) spectra of Py–Th samples. Pristine (black line) and samples after immersion in HCl aqueous solution. (inset) photograph of Py–Th nanosheets dispersion in HCl aq.

The anisotropic morphology of Py–Th was observed by scanning electron microscopy (SEM). The average particle size of Py–Th was 6.51 ± 3.0 μm. The Py–Th particle exhibited different surface morphologies: smooth and pleated in the plane and vertical directions of the image, respectively. The pleated surface corresponded to a cross-section of the laminated layers (highlighted by red arrow in Fig. 4a). The π–π stacking of the primal 2D triazine networks of Py–Th formed the layered structure on the micrometer scale. Mesopore and micropore of Py–Th were not observed by typical nitrogen adsorption method. Its amorphous state in the lateral direction may inhibit to constructing construct continuous pores. The conductivity of Py–Th was measured on the pelletized samples, but it did not show a significant value. The behaviour should be originating from its semiconductor type electronic structure according to the previous literatures.^2,3^

The layered Py–Th particle was exfoliated into nanosheets in the liquid phase (Fig. 4b). The layered Py–Th was dispersed in an HCl aqueous solution (1 mol dm^−3^) under ultrasonic irradiation for 15 min. Thereafter, the dispersion liquid was maintained under stirring at 60 °C for 24 h. The dispersion of Py–Th was obtained after centrifugation and decantation to remove the unexfoliated precipitate. The SEM images show the Py–Th nanosheets, which exhibited an anisotropic shape whose size was 2.23 ± 0.30 μm in width and 103 ± 20 nm in thickness (Fig. 4c and d).

The efficiency of the exfoliation was estimated from the weight of the precipitate in the centrifugation step. The exfoliation yields of Py–Th and Py were 80% and 66%, respectively, which were comparable to those of earlier studies under harsh conditions, such as mechanical mixing and chemical oxidation.^32–36^ The driving force of the exfoliation process was considered to be the protonation of the pyridine moieties under acidic conditions. The protonation of Py–Th was confirmed by UV-Vis spectroscopy (Fig. 3e). The Py–Th spectrum exhibited a redshift of its absorption edge after it had been immersed in an aqueous HCl solution. The redshift was not observed for Ph–Th but for Py (Fig. S2†). The protonated pyridine moieties facilitated the intercalation of solvent molecules. In addition, the low crystallinity of Py–Th may have prompted the exfoliation according to the difference in the yields of Py–Th and Py.

### 3.4 Electrochemical properties

**Fig. 5:**
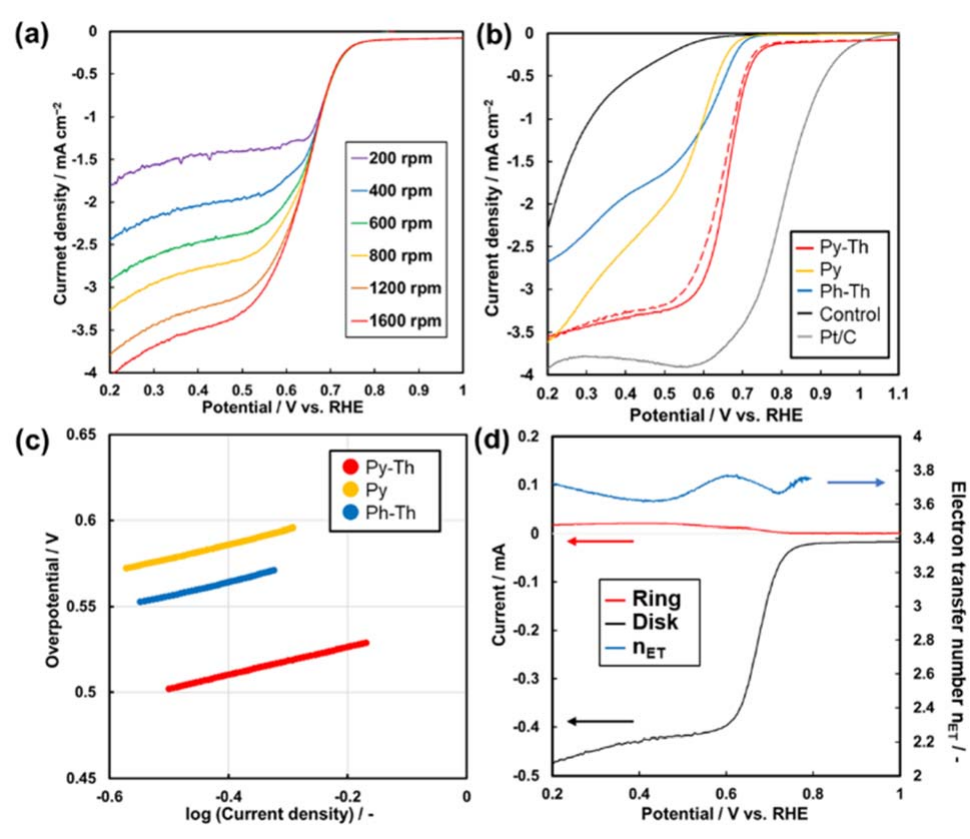
Electrocatalytic ORR properties of Py–Th and related samples in O_2_ saturated 0.1 mol dm^−3^ KOH aqueous electrolyte. (a) LSV of Py–Th on various rotating speed. (b) LSV of Py–Th (red line), Ph–Th (blue line), Py (yellow line), the control samples consisting of conductive carbon and binder (black line), Pt/C (grey line), and layered Py–Th after cyclic voltammetry in the range 1.2 to 0 V (*vs.* RHE) for 250 cycles (red dashed line). RDE working electrode was set to be 1600 rpm of rotating speed. (c) Tafel plots corresponding to the LSV (d) relationship of disk current on Py–Th electrode (black line), ring current (red line), and calculated *n*_ET_ (blue line). RRDE Pt ring electrode was set to 0.3 V *vs.* RHE.

The Py–Th electrode functioned as an ORR electrocatalyst in a basic aqueous electrolyte (Fig. 5a and S4†). The working electrode was prepared by the proposed polymerization method in the presence of conductive carbons as supports. The dispersion of the mixture comprising the triazine networks, conductive carbon, and binder ionomer was cast on a glassy carbon electrode. The linear sweep voltammetry (LSV) results of the triazine network electrodes indicated that the Py–Th electrode exhibited a superior electrocatalytic ORR performance than those of Py and Ph–Th (Fig. 5b). The onset potentials of the Py–Th, Py, and Ph–Th electrodes were 0.76, 0.66, and 0.69 V (*vs.* RHE), respectively. The LSV results of the Py–Th electrode exhibited a diffusion-limited current in a high overpotential region, while those of the Ph–Th and Py electrodes exhibited a reaction-limited current mode. The half-wave potential was 0.68 V *vs.* RHE on the Py–Th electrode, and its catalytic property was reproducible (Fig. S5†). The Tafel slopes were 81, 84, and 85 mV per decade for Py–Th, Py, and Ph–Th, respectively (Fig. 5c). Moreover, the onset potential of Py–Th was unchanged after cyclic voltammetry within 1.2–0 V (*vs.* RHE) for 250 cycles (red dashed lines, Fig. 5b). The activity of the Py–Th electrode was hardly changed when electrolyte contained methanol (Fig. S5†). These results implied that Py–Th can be used as a superior ORR electrocatalyst.

The selectivity of the 4-electron reduction path and 2-electron reduction path was studied by using rotated ring-disk type working electrode (Fig. 5d). The estimated electron transfer number is 3.77 for Py–Th electrode at 0.60 V *vs.* RHE. The value indicates that the 4-electron reaction is a major path. The enhanced electrocatalytic performance was explained by the combination of pyridine ring and thiophene ring in flat conjugated plane. A number of the previous works proposed that the active site of electrocatalytic ORR was the carbon atom next to pyridinic nitrogen (Fig. S6†).^22,41,42^ The electron donating thiophene moiety contributes to increasing Lewis basicity of the active site. Coexistence of both electron donor and active site in planer CTNs is effective design for enhanced organic ORR catalyst.

The onset potential of Py–Th is one of its best properties, compared with previous studies regarding the structure-defined covalent organic frameworks (Fig. S7 and Table S2†). In terms of the half-wave potential and the *n*_ET_, Py–Th showed properties that were comparable to those of the top groups. Although a few previous studies reported superior properties, they employed a high-temperature process that degraded the molecular structure. The Py–Th electrode exhibited enhanced properties and stability as a metal-free and structure-defined ORR electrocatalyst. Further enhancement of the catalytic performance can be achieved using an advanced structural design from molecular to nanometer scales.

## 4. Conclusions

The synthesis and electrocatalytic application of amorphous layered triazine networks were developed. The distortion-free combination of heteroaromatic rings was the key strategy for the layered structure. The nanosheets were easily obtained using the protonation-assisted exfoliation method. The laterally low-periodic layered triazine networks exhibited enhanced ORR electrocatalytic activities. These networks, positioned between covalent triazine frameworks and CTN, contribute to expanding the molecular design of organic 2D materials toward high-performance electrocatalysts.

## Conflicts of interest

There are no conflicts to declare.

## Supplementary Material

RA-013-D3RA01431B-s001
