# Supplementary material for: Thienylene combined with pyridylene through planar triazine networks for applications as organic oxygen reduction reaction electrocatalysts
Source: RSC Adv. 2023 Apr 17;13(17):11794–9. doi: 10.1039/d3ra01431b (PMC10107030; doi:10.1039/d3ra01431b)
Supplement: RA-013-D3RA01431B-s001 [file RA-013-D3RA01431B-s001.pdf]

Electronic Supplementary Material (ESI) for RSC Advances.
This journal is © The Royal Society of Chemistry 2023

Electronic Supplementary Information

Thienylene combined with pyridylene through triazine networks for applications as organic oxygen reduction reaction electrocatalysts

Kosuke Sato*, Nodoka Osada, Hidenori Aihara

Organic Materials Chemistry Group, Sagami Chemical Research Institute

2743-1 Hayakawa, Ayase, Kanagawa 252-1193, Japan

E-mail: kosuke-sato@sagami.or.jp

Contents

Detailed experimental procedure	P. S2
Synthesis methods (Scheme S1, S2 and S3)	P. S4
Attribution of ^{13}C-NMR chart and FT-IR spectrum of **Py-Th** (Fig. S1)	P. S6
Characterization of **Py** and **Ph-Th** (Fig. S2)	P. S7
Calculated conformations of the CTNs (Fig. S3 and Table S1)	P. S8
Koutecký-Levich plots of the LSV measurements (Fig. S4)	P. S10
The electrochemical ORR performances on the **Py-Th** electrode (Fig. S5)	P. S11
DFT calculation about the active site on **Py-Th** (Fig. S6)	P. S12
Comparison of the ORR properties with previous reports. (Fig. S7 and Table S2)	P. S13
Supporting references	P. S15

Detailed experimental procedure

Exfoliation of the triazine networks.

The CTNs samples (30 mg) and 1.0 mol L^{-1} HCl aqueous solution (10 mL) were mixed and sonicated for 15 min. The mixture was agitated by magnetic stirrer at 60 °C for 24 h. After cooling to the room temperature, the mixture was centrifuged at 2000 rpm for 10 min. The dispersion was separated by decantation and the precipitates were corrected and dried. The exfoliation yield is calculated based on eq-(1). W_{initiqal} was 30 mg and $W_{\text{precipitate}}$ was weight of the corrected precipitates.

$$(Exfoliation\ yield) = \frac{W_{initial} - W_{precipitate}}{W_{initial}} \quad \ldots \textbf{eq-(1)}$$

Electrochemical measurements.

Linear sweep voltammetry was performed by a potentiostat (BioLogic, VSP-300) at scan rate 10 mV sec^{-1}. 0.1 mol L^{-1} KOH solution was used as electrolyte. To saturate the electrolyte with O_2, the electrolyte was bubbled with O_2 for 10 min prior to each experiment and O_2 was flowed through the electrolyte during the measurements. The conversion factors for Ag/AgCl to RHE are 0.958 V in 0.1 mol L^{-1} KOH solution according to eq-(2). The pH value of the electrolyte was measured by pH meter (HORIBA, F-71S).

$$E_{RHE} - E_{Ag/AgCl} = 0.059 \times pH + 0.199 \quad \ldots \textbf{eq-(2)}$$

Rotated ring disc electrode (RRDE, PINE Research, AFE6R2GCPT) is used in the measurement to estimate the electron transfer number. In the LSV measurement, the RRDE was rotated at 400 rpm and the ring electrode (Pt) was maintained at −0.1 V vs. Ag/AgCl. The electron transfer number (n_{ET}) is calculated based on eq-(3). The collection efficiency (E_{collect}) is 38.3% for the used RRDE.

$$n_{ET} = \frac{4 \times I_{disk}}{I_{disk} + \left({I_{ring}}/{E_{collect}} \right)} \quad \ldots \textbf{eq-(3)}$$

Scheme S1 Synthesis of **Py-Th**

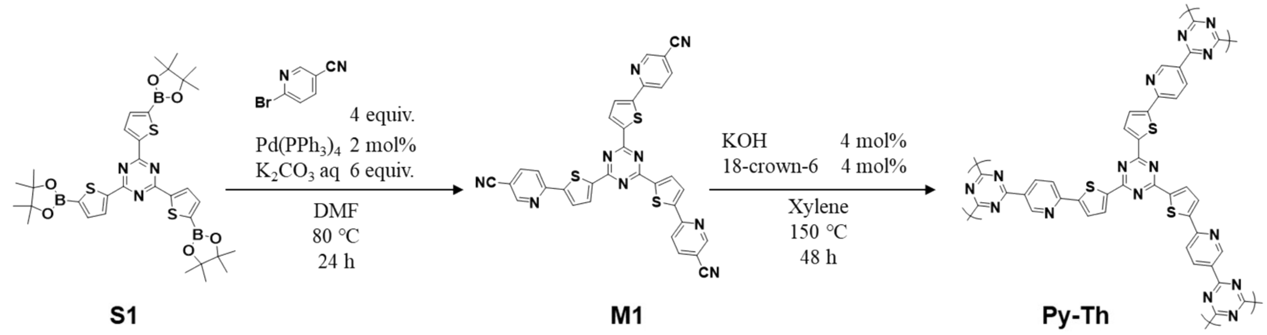

M1. Tris[2-(4,4,5,5-tetramethyl-dioxaborroran-2-iyl)-thiphene-5-iyl]-1,3,5-triazine (**S1**) was synthesized via known method reported in the previous work.[s1] 2-bromo-5-cyano-pyridine (98%) was purchased from TCI chemical. Under argon atmosphere, **S1** (100 mg, 0.15 mmol), 2-bromo-5-cyano-pyridine (110 mg, 0.60 mmol), and tetra(triphenylphosphine)palladium (2 mg, 0.002 mmol) was suspended in DMF (5 mL). To the suspension was added potassium carbonate aqueous solution (0.2 mol L^{-1}, 6 mL) and the mixture was stirred at 80 °C for 24 h. After cooling to the room temperature, water and ethyl acetate were added to the mixture. The crude product was corrected by filtration. The crude product was purified by recrystallization from 1,2-dichlorobenzene to give [2-(5-cyano-pyridine-2-iyl)-thiophene-5-iyl]-1,3,5-triazine (**M1**) as yellow solid (45 mg, 0.08 mmol, 48 %).

^{1}H-NMR($CDCl_3$): δ= 7.65 (d, J=5.0 Hz, 3H), 7.80(d, J= 8.8 Hz, 3H), 7.86(dd, J=8.8, 1.3 Hz, 3H), 8.31(d, J=5.0 Hz, 3H), 8.89 (d, J=1.3Hz, 3H) ppm.

Py-Th. Under air, potassium hydroxide (1.9 mg, 0.033 mmol) and 18-crown-6 ether (8.5 mg, 0.033 mmol) was dissolved in ethanol (1 mL) and stirred for 10 min. The solution was concentrated to remove solvent under reduced pressure to give an oil product. To this oil, **M1** (500 mg, 0.80 mmol) and xylene (0.8 mL) were added. The mixture was refluxed at 150°C for 48 h. After cooling to the room temperature, the crude product was corrected by filtration. The crude product was purified by washing with xylene, $CHCl_3$ and water to give **Py-Th** as yellow solid (405 mg, 81%).

Scheme S2 Synthesis of **Py**

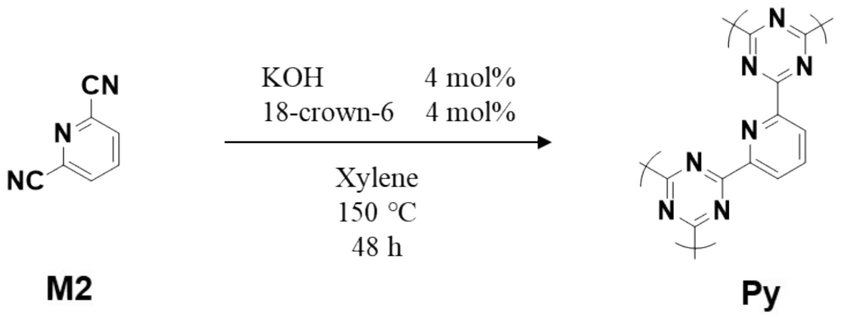

Py. 2,6-dicyano-pyridine (**M2**, purity ~98%) was purchased from Sigma-Aldrich. Under air, potassium hydroxide (1.9 mg, 0.033 mmol) and 18-crown-6 ether (8.5 mg, 0.033 mmol) was dissolved in ethanol (1 mL) and stirred for 10 min. The solution was concentrated to remove solvent under reduced pressure to give an oil product. To this oil, **M2** (103 mg, 0.80 mmol) and xylene (0.8 mL) were added. The mixture was refluxed at 150°C for 48 h. After cooling to the room temperature, the crude product was corrected by filtration. The crude product was purified by washing with xylene, $CHCl_3$ and water to give **Py** as gray solid (55 mg, 53%).

Scheme S3 Synthesis of **Ph-Th**

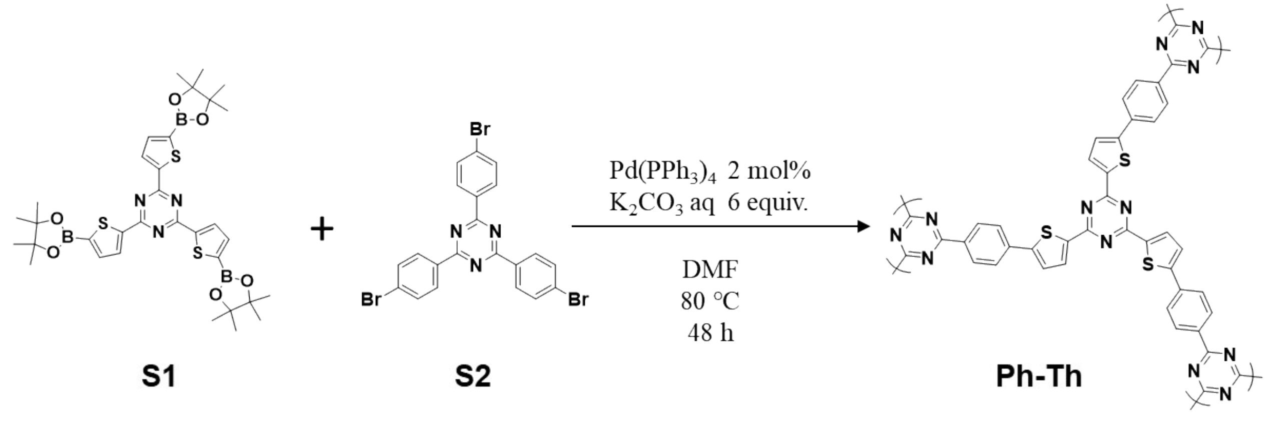

Ph-Th. Tri(4-bromo-phenyl)-1,3,5-triazine (**S2**) was synthesized via known method reported in the previous work.[s2] Under argon atmosphere, **S1** (100 mg, 0.152 mmol), **S2** (85 mg, 0.152 mmol) and tetra(triphenylphosphine)palladium (2 mg, 0.002 mmol) was suspended in DMF (4 mL). To the suspension was added potassium carbonate aqueous solution (0.2 mol L^{-1}, 6 mL) and the mixture was stirred at 150 °C for 24 h. After cooling to the room temperature, the crude product was corrected by filtration. The crude product was purified by washing with $CHCl_3$, DMF and water to give **Ph-Th** as yellow solid (60 mg, 66 %).

Attribution of ^{13}C-NMR chart and FT-IR spectrum of Py-Th (Fig. S1)

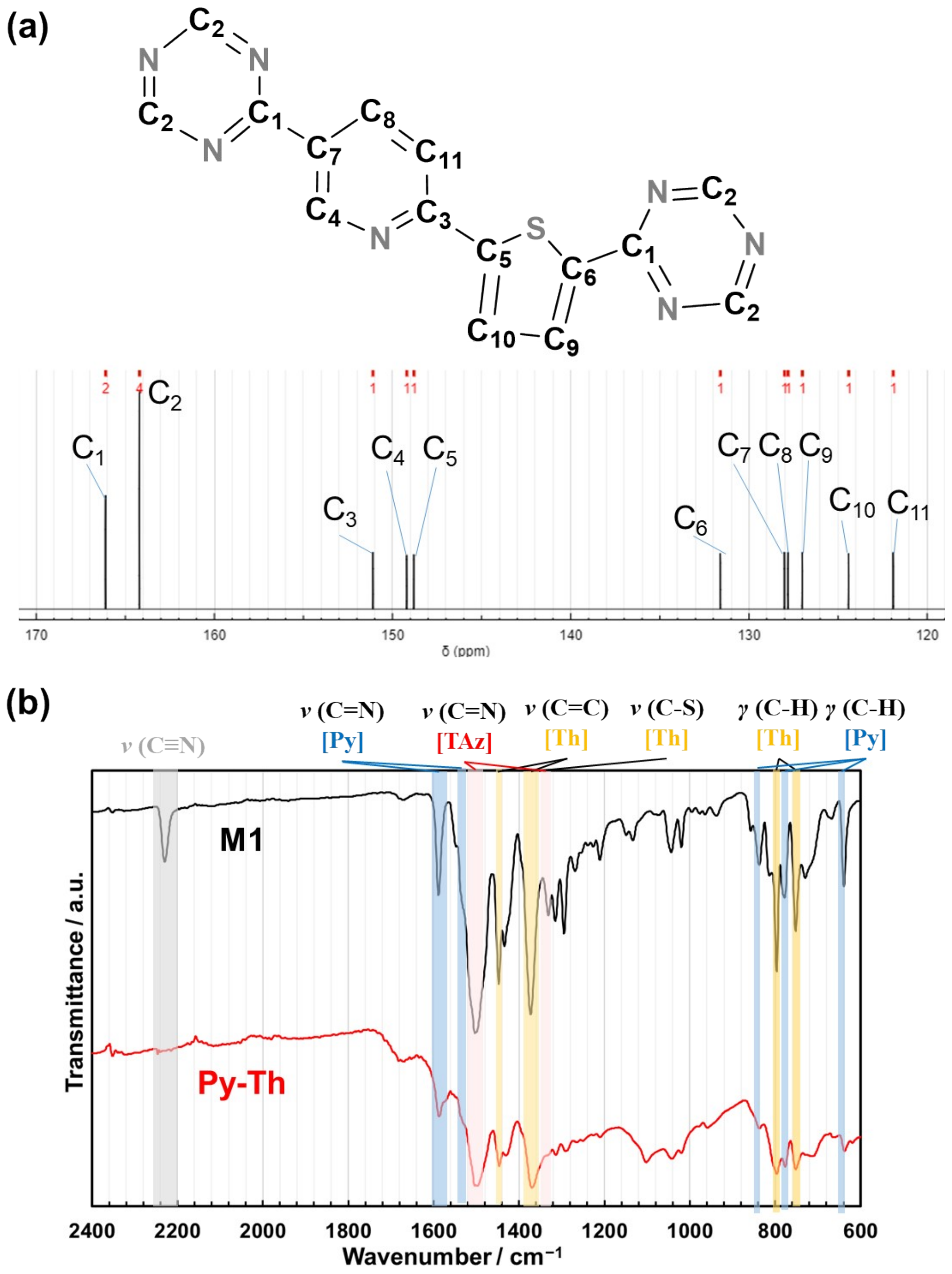

Fig. S1. (a)Simulated ^{13}C-NMR spectrum of the simplified structure originating to **Py-Th**. The simulation was performed by NMRDB[s3]. (b) Detailed peak attribution of FT-IR spectra.

Characterization of Py and Ph-Th (Fig. S2)

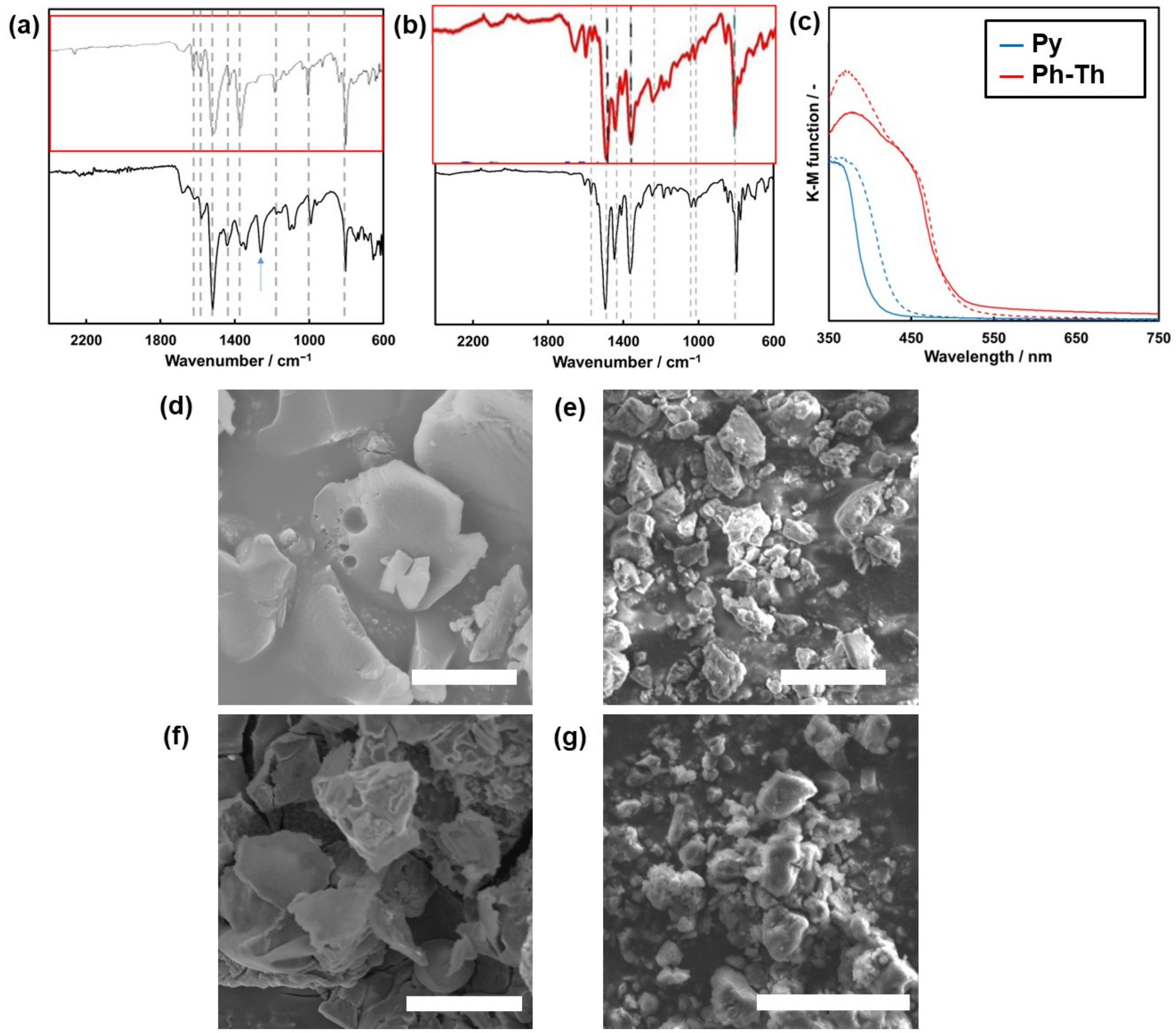

Fig. S2. (a) FT-IR spectra of Py and that from literature (region in red square).[s4a] The absorption peak pointed by blue arrow was derived from pyridyl amide moiety that should be generated from the terminal nitril group and KOH. (b) FT-IR spectra of Ph-Th and that from literature (region in red square).[s4b] (c) UV-vis spectra of **Py** (blue line), **Ph-Th** (red line) and the acid treated samples (corresponding hashed lines). (d,e) SEM images of **Py** (d), **Ph-Th** (e). The inserted scale bar is 1 μm and 5μm, respectively. (f,g) SEM images of **Py** (f), **Ph-Th** (g) after HCl immersion and sonication. The inserted scale bar is 1 μm and 5 μm, respectively.

Calculated conformations of the CTNs (Fig. S3 and Table S1)

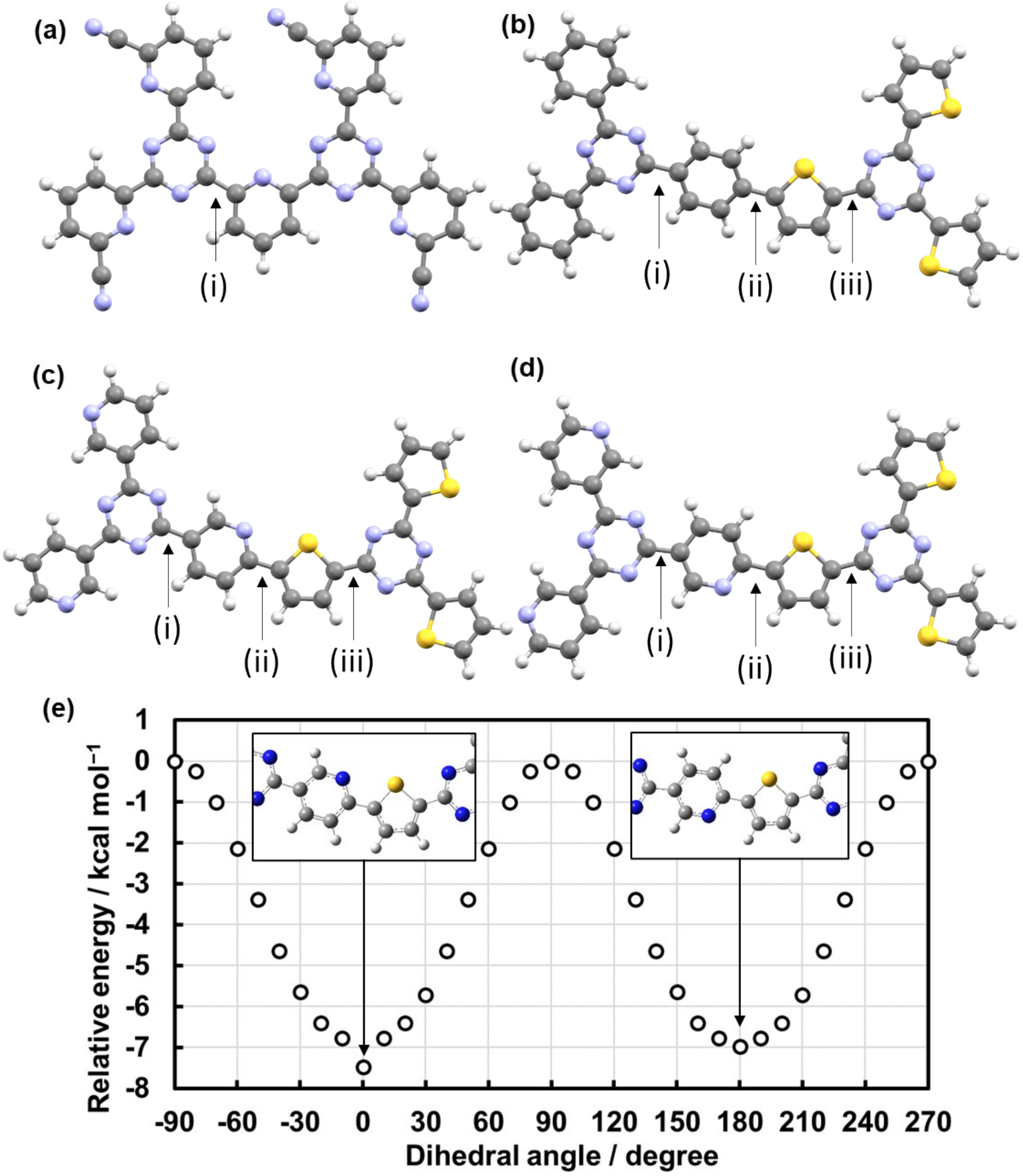

Fig. S3. (a–d) Optimized structures of model compound for **Py** (a), **Ph-Th** (b), **Py-Th** (c,d). DFT calculations were performed based on B3LYP-D3BJ/6-31G++(d,p) level theory by using Gaussian 16.[s5] (e) Relationship of dihedral angle and relative energy of **Py-Th** describing the rotation around the bond between the pyridine ring and the thiophene ring.The model compound of **Py-Th** shows two different stable conformations with high planarity.

Table S1 The dihedral angles pointed in Fig. S3.

	polymer	(i)	(ii)	(iii)
(a)	**Py**	0.75°	–	–
(b)	**Ph-Th**	0.02°	24.5°	0.00°
(c)	**Py-Th**	0.01°	0.00°	0.00°
(d)	**Py-Th**	0.01°	0.00°	0.00°

Koutecký-Levich plots of the LSV measurements (Fig. S4)

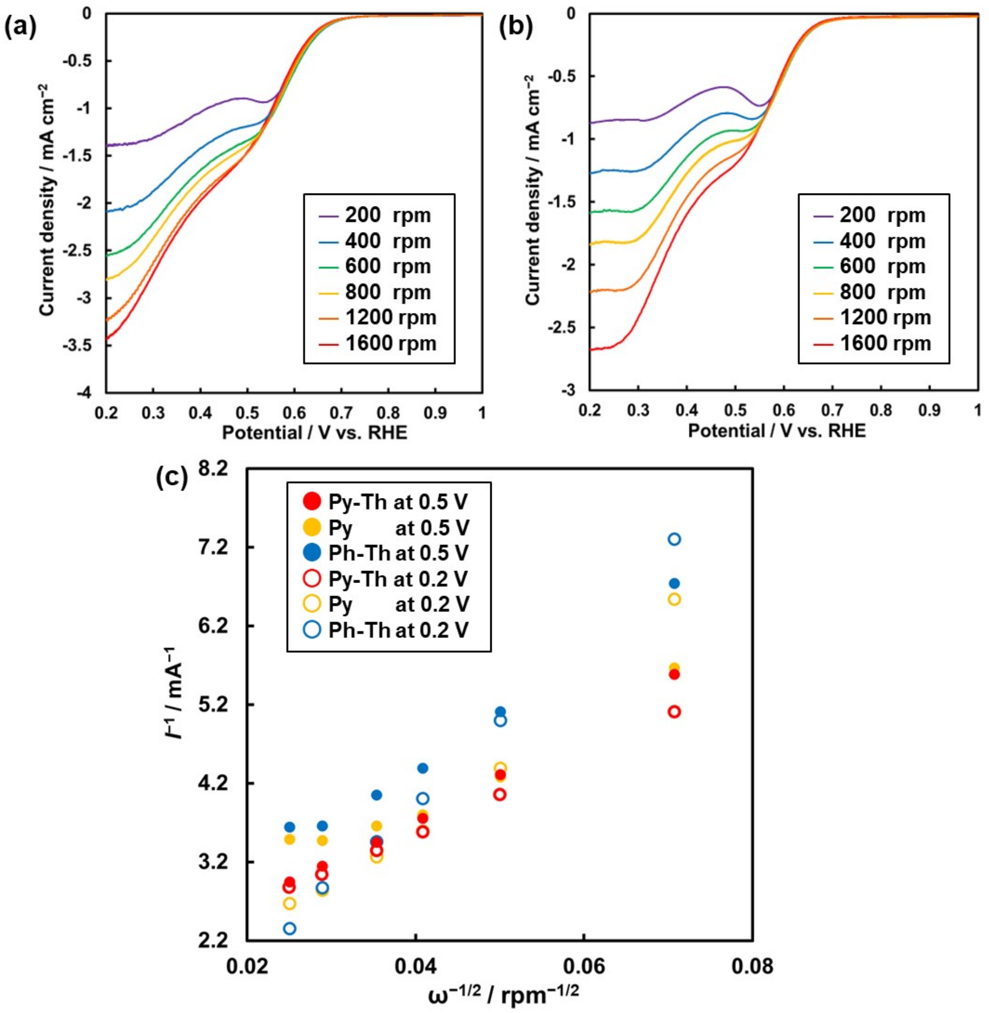

Fig. S4 (a,b) LSV of the **Py** electrode (a) and the **Ph-Th** electrode (b) on various rotating speed. (c) K-L plot chart corresponding to the RDE measurements.

According to the trend of the K-L plot, the calculated electron number was 3.32, 3.01, and 2.49 for **Py-Th**, **Py**, and **Ph-Th**, respectively. Following values in (eq.4) were from literature.[s6] *D*: the diffusion coefficient of O_2 in 1 mol L^{-1} KOH solution (1.76×10^{-5} cm^2 s^{-1}), *v*: the kinetic viscosity of the water (0.01 cm^2 s^{-1}), *C*: the concentration of O_2 in 1 mol L^{-1} KOH solution at 25 °C (1.103×10^{-6} mol cm^{-3}).

$$I = 0.620nFAD^{2/3}\omega^{1/2}\nu^{-1/6}C \quad ...(eq.4)$$

The electrochemical ORR performances on the Py-Th electrode (Fig. S5)

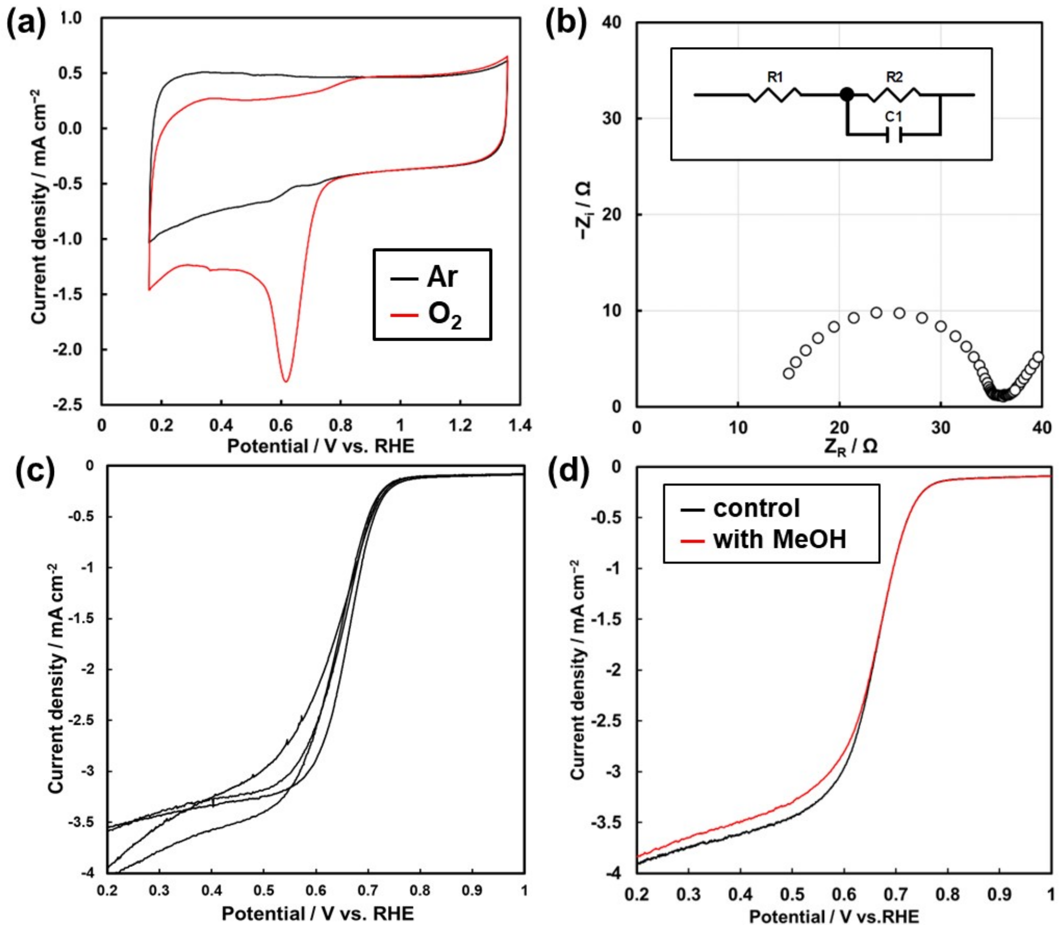

Fig. S5 (a) CV on **Py-Th** electrode in 0.1M KOH electrolyte under Ar and O_2 gas flow. Sweep rate was set to 50 mV/sec. (b) Nyquist plot of **Py-Th** electrode. The EIS measurement was performed at 0.60 V vs. RHE with 10 mV of amplitude in frequency range from 2 MHz to 0.2 Hz. Inset: the equivalent circuit used for fitting. The simulated values are 14.7 Ω for R1; 20.1 Ω for R2; 18.7 nF for C1. (c) LSV of the 4 different **Py-Th** samples in O_2 saturated 0.1M KOH electrolyte with 1600 rpm of rotating speed. LSV was measured with 10 mV sec^{-1} of sweep rate and from 0.4 V to −0.8 V vs. Ag/AgCl in potential range. (d) LSV for methanol tolerance test performed in O_2 saturated 0.1 M KOH with 1 M MeOH aqueous solution.

The measured catalytic properties were reproducible. The slight differences in the mixed and coating states of the samples caused the data variation especially in the higher overpotential region.

DFT calculation about the active site on Py-Th(Fig.S6)

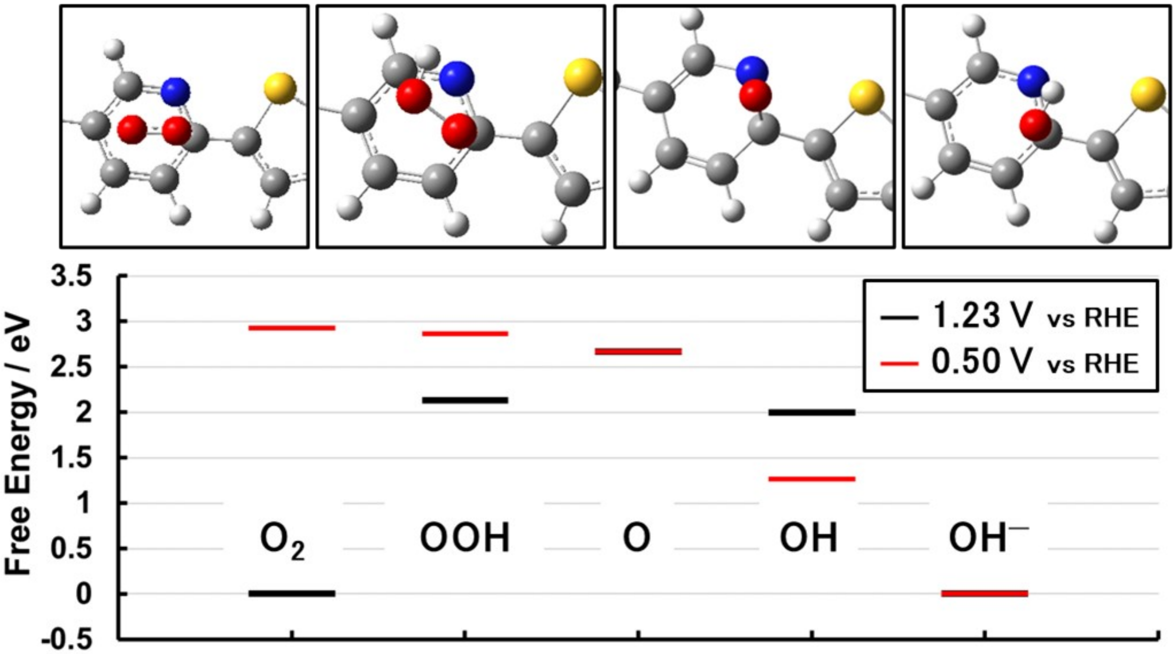

Fig. S6 DFT calculation of estimated reaction coordinate on ORR. Every intermediates derived from model compounds were optimized on B3LYP+g3bj/6-31++g(d,p) level theory. The free energy diagram was calculated from the Gibbs energy of the intermediates, reported value from literature, and electron potential according to following eq (4−7).[s6]

$$\Delta G_{O_2\_OOH} = G_{OOH*} + G_{H_2O} - G_{cat} - 3G_{OH^-} - 3eU \quad \text{… eq-(4)}$$

$$\Delta G_{OOH\_O} = G_{O*} - G_{OOH*} + G_{OH^-} - eU \quad \text{… eq-(5)}$$

$$\Delta G_{O\_OH} = G_{OH*} - G_{O*} - G_{H_2O} + G_{OH^-} - eU \quad \text{… eq-(6)}$$

$$\Delta G_{OH\_OH^-} = - G_{OH*} + G_{cat} + G_{OH^-} + eU \quad \text{… eq-(7)}$$

The O_2 reduction path on **Py-Th** can be simulated on the model compound. The lack of consideration about the periodicity and the solvent effect might result in the overestimation of the overpotential at which this reaction system reached the downhill mode.

Comparison of the ORR properties with previous reports. (Table S2 and Fig. S7)

Table S2 Relationships of onset potential and electron transfer number on ORR among previously reported structure-defined organic electrode.

**ID**	**Plot color**	name	**Onset potential V vs. RHE**	**Half-wave potential V vs. RHE**	**Diffusion-Limit**	**n**	**Ref.**
**1**	●	CTF-CSU19	**0.7**	**0.61**	-	**2.64**	[19]
**1**	●	CTF-CSU1	**0.75**	**0.63**	-	**2.6**	[19]
**2**	●	TFPB-TAPB-COF	**0.72**	**0.63**	+	**2.74**	[20]
**2**	●	BTT-TAPB-COF	**0.79**	**0.71**	+	**3.53**	[20]
**2**	●	BTT-TAT-COF	**0.82**	**0.75**	+	**3.66**	[20]
**3**	●	Porphyrin-COF	**0.81**	**0.70**	+	**3.7**	[14]
**5**	●	TPA-BP-1	**0.72**	**0.62**	-	**4†**	[16]
**5**	●	TPA-TPE-2	**0.69**	**0.60**	-	**4†**	[16]
**6**	-	TPA-COF	**0.70**	**0.60**	-	**N.A.**	[22]
**6**	-	DFP	**0.70**	**0.60**	+	**N.A.**	[22]
**6**	-	TAPT	**0.74**	**0.63**	-	**N.A.**	[22]
**6**	●	Taz-COP	**0.77**	**0.68**	-	**3.52**	[22]
**7**	●	MCAC	**0.83**	**0.75**	+	**3.9**	[21]
**8**	●	Th-TPB-COF1	**0.71**	**0.62**	+	**2.08**	[17]
**8**	●	Th-TPB-COF2	**0.75**	**0.66**	+	**3.46**	[17]
**8**	●	Th-TPB-COF3	**0.80**	**0.72**	+	**3.81**	[17]
**This work**	●	Py-Th	**0.77**	**0.69**	+	**3.77**	

† these electron transfer number were estimated based on RDE measurement and Levich equation.The electrocatalytic ORR properties of **Py-Th** are comparable to that of the previous reports. Even though some previous works reported the superior properties, those samples were synthesized in higher temperature or contain unstable imine groups for polymerization.

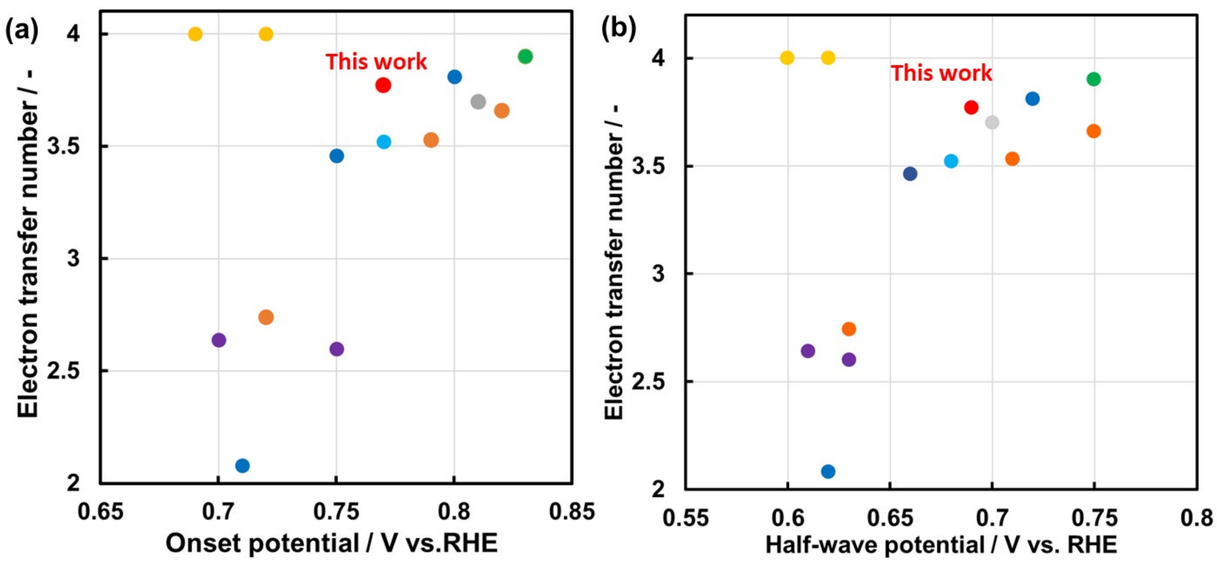

Fig. S7 (a) Relationships of the onset potential and electron transfer number on ORR among previously reported structure-defined organic electrode. (b) Relationships of the half-wave potential and electron transfer number on ORR.

Supporting references

[s1] A. Naka, R. Fukuda, R. Kishimoto, Y. Yamashita, Y. Ooyama, J. Ohshita and M. Ishikawa, *J. Organomet. Chem.*, 2012, **702**, 67–72.

[s2] M. R. Liebl and J. Senker, *Chem. Mater.*, 2013, **25**, 970–980.

[s3] www.nmrdb.org: Resurrecting and processing NMR spectra on-line

Banfi and L. Patiny, *Chimia*, 2008, **62**, 280–281.

[s4] (a) P. Kuhn, M. Antonietti and A. Thomas, *Angew. Chem. Int. Ed.*, 2008, **47**, 3450–3453.

(b) W. Huang, J. Byun, I. Rörich, C. Ramanan, P. W. M. Blom, H. Lu, D. Wang, L. C. da Silva, R. Li, L. Wang, K. Landfester and K. A. I. Zhang, *Angew. Chem. Int. Ed.*, 2018, **57**, 8316–8320.

[s5] Gaussian 16, Revision C.01

M. J. Frisch, G. W. Trucks, H. B. Schlegel, G. E. Scuseria, M. A. Robb, J. R. Cheeseman, G. Scalmani, V. Barone, G. A. Petersson, H. Nakatsuji, X. Li, M. Caricato, A. V. Marenich, J. Bloino, B. G. Janesko, R. Gomperts, B. Mennucci, H. P. Hratchian, J. V. Ortiz, A. F. Izmaylov, J. L. Sonnenberg, D. Williams-Young, F. Ding, F. Lipparini, F. Egidi, J. Goings, B. Peng, A. Petrone, T. Henderson, D. Ranasinghe, V. G. Zakrzewski, J. Gao, N. Rega, G. Zheng, W. Liang, M. Hada, M. Ehara, K. Toyota, R. Fukuda, J. Hasegawa, M. Ishida, T. Nakajima, Y. Honda, O. Kitao, H. Nakai, T. Vreven, K. Throssell, J. A. Montgomery, Jr., J. E. Peralta, F. Ogliaro, M. J. Bearpark, J. J. Heyd, E. N. Brothers, K. N. Kudin, V. N. Staroverov, T. A. Keith, R. Kobayashi, J. Normand, K. Raghavachari, A. P. Rendell, J. C. Burant, S. S. Iyengar, J. Tomasi, M. Cossi, J. M. Millam, M. Klene, C. Adamo, R. Cammi, J. W. Ochterski, R. L. Martin, K. Morokuma, O. Farkas, J. B. Foresman, and D. J. Fox, Gaussian, Inc., Wallingford CT, 2019.

[s6] H. Meng and P. K. Shen, *Electrochem. Commun.*, 2006, **8**, 588–594.

[s7] (a) Q. Lv, W. Si, J. He, L. Sun, C. Zhang, N. Wang, Z. Yang, X. Li, X. Wang, W. Deng, Y. Long, C. Huang and Y. Li, *Nat. Commun.*, 2018, **9**, 3376. (b) Y. Jiao, Y. Zheng, M. Jaroniec and S. Z. Qiao, *J. Am. Chem. Soc.*, 2014, **136**, 4394–4403.

<Previous reports studying organic electrodes for ORR>

[14] J. Guo, C. Lin, Z. Xia and Z. Xiang, *Angew. Chem. Int. Ed.*, 2018, **57**, 12567–12572.

[16] S. Roy, A. Bandyopadhyay, M. Das, P. P. Ray, S. K. Pati and T. K. Maji, *J. Mater. Chem. A*, 2018, **6**, 5587–5591.

[17] D. Li, C. Li, L. Zhang, H. Li, L. Zhu, D. Yang, Q. Fang, S. Qiu and X. Yao, *J. Am. Chem. Soc.*, 2020, **142**, 8104–8108.

[19] W. Yu, S. Gu, Y. Fu, S. Xiong, C. Pan, Y. Liu and G. Yu, *J. Catal.*, 2018, **362**, 1–9.

[20] G. Jiang, L. Zhang, W. Zou, W. Zhang, X. Wang, H. Song, Z. Cui and L. Du, *Chinese J. Catal.*, 2022, **43**, 1042–1048.

[21] J. Liu, C. Wang, Y. Song, S. Zhang, Z. Zhang, L. He and M. Du, *J. Colloid Interface Sci.*, 2021, **591**, 253–263.

[22] T. Boruah, S. K. Das, G. Kumar, S. Mondal and R. S. Dey, *Chem. Commun.*, 2022, **58**, 5506-5509.
